# A new era of medical therapy for peripheral artery disease

**DOI:** 10.1590/1677-5449.190056

**Published:** 2020-05-08

**Authors:** Ivan Benaduce Casella, Calógero Presti

**Affiliations:** 1 Universidade de São Paulo – USP, Faculdade de Medicina, Hospital das Clínicas, Disciplina de Cirurgia Vascular, São Paulo, SP, Brasil.

Until recently, medical therapy has always played a secondary role in the management of symptomatic peripheral artery disease, focusing on control of risk factors for atherosclerosis. Strict medical treatment had limited results in avoiding clinical outcomes caused by the evolution of atherosclerotic disease.^1^^,^^2^ However, clinical investigations concluded in the recent years opened new possibilities for medical therapy that goes far beyond the former role of an adjuvant therapy. Some are already a reality incorporated in daily practice, while are still under investigation.

## STATINS

For more than two decades, statins have assumed an importance in the clinical management of atherosclerotic disease that exceeds its role in lipid control. Since its early use, the pleiotropic effects of this class of medications have been documented, either by i*n vitro* or clinical evidence.

Among the most desirable pleiotropic effects of statins in PAD we can list the reduction of platelet activation, endothelial disfunction and inflammatory responses related to the progress of atherothrombotic disease.^3^^-^5 Statins promote benefic changes in atherosclerotic plaque composition, reducing their necrotic core and total atheroma volume.^6^

Statins also have an important influence in the physiology of coagulation, decreasing thrombin generation and enhancing thrombomodulin expression which results in a synergic effect over endogenous anticoagulant processes.^7^

The clinical benefits of statin therapy for patients going under major non-cardiac arterial surgery was well documented by Durazzo et al. In a prospective, double-blind trial comparing perioperative use of atorvastatin versus placebo, the authors observed a 18% absolute reduction in the composite outcome of death from cardiovascular causes, acute myocardial infarction, ischemic stroke and unstable angina for the statin group.^8^

## RIVAROXABAN

Rivaroxaban is a direct oral anticoagulant that inhibits Factor Xa. Although a relatively new drug, has gained wide use in the treatment of venous thromboembolism and for stroke prevention in non-valvar atrial fibrillation.

Recent investigations also showed the role of rivaroxaban in the prevention of cardiovascular events in patients with stable cardiovascular disease. The COMPASS study^9^ compared rivaroxaban, either as a single therapy (5mg twice daily) or associated to a platelet aggregation inhibitor (Rivaroxaban 2.5mg twice daily + ASA 100mg once daily) with the gold-standard therapy (ASA 100mg once daily). The inclusion criteria were, in a simplified description, symptomatic stable coronary and/ or peripheral vascular disease with additional risk factors (smoking, diabetes and others). With a 23-month mean follow-up, the combined therapy of low-dose rivaroxaban plus aspirin reduced the combined outcomes of cardiovascular death, myocardial infarction and ischemic stroke in 24%. An increased risk of major bleeding was observed in the rivaroxaban+ASA group, without observed differences in the number of fatal bleedings between the groups.

The COMPASS PAD sub-study^10^ focused in 7000+ patients with stable peripheral artery disease (mostly lower limbs and carotid disease) from the original compass study. The combination Rivaroxaban + ASA repeated the benefits of the main investigation, with a 28% decrease in major cardiovascular outcomes compared with the ASA monotherapy group. Also, direct benefits related to the lower limbs were observed, with a 46% reduction on major adverse limb events (MALE - development/progress of acute limb ischemia, chronic critical limb ischemia, amputations) and 70% reduction in major amputations.

## MONOCLONAL ANTIBODIES

Monoclonal antibodies (MABs) are one of the leading innovations in medical therapy for several clinical specialties. More than 500 MABs are already approved or under investigation for medical use. Many of these have effect over cardiovascular and peripheral vascular disease. Some of the most promising members of this pharmacologic class are briefly described below.

Tocilizumab is a drug that targets interleukin-6 and is primarily used for therapy of several rheumatologic disorders. Recently, FDA has approved tocilizumab as a treatment for giant cell arteritis. The combined use of tocilizumab plus prednisone for 26 weeks resulted in a 53 to 56% sustained remission (absence of giant-cell arteritis signs and symptoms, erythrocyte sedimentation rate < 30 mm/h, C-reactive protein < 1 mg/dL) rate in 52 weeks against a 14-18% remission rate for the prednisone plus placebo group.^11^ Tocilizumab has also showed benefits in improving endothelial function and reducing aortic stiffness in patients with rheumatoid arthritis,^12^ but no studies were specifically directed to atherosclerotic PAD patients.

Evolocumab is a proprotein convertase subtilisin-kexin type 9 (PCSK9) inhibitor that leads to an expressive reduction in LDL-Cholesterol plasma levels. The Fourier clinical trial^13^ tested a combination of evolocumab plus statins compared with placebo plus statins in 27,000 + patients with previously reported cardiovascular disease. The study pointed a 15% reduction of composite cardiovascular events (cardiovascular death, myocardial infarction, stroke, unstable angina and myocardial revascularization) in 26 months for patients treated with evolocumab, with a low incidence of adverse events for both groups. The effects of evolocumab were more expressive in PAD patients, as observed in a specific substudy^14^ directed to the lower extremity arterial disease population (3642 individuals), with a 21% reduction of the composite outcome of cardiovascular events. The incidence of major adverse limb events for evolocumab-treated patients was also reduced in 42% when compared to placebo-treated individuals.

Interleukin-1β is strongly related to inflammatory processes, as well as atherosclerosis formation and progression. Canakinumab is an interleukin-1β inhibitor, tested in a prospective, double blind trial against placebo for patients with previous myocardial infarction and persistent inflammatory activity defined by high-sensitivity C-reactive protein levels equal or above 2mg/L. The primary efficacy end point was a composite of cardiovascular events (nonfatal myocardial infarction, nonfatal stroke, cardiovascular death). There was a significant (14-15%) reduction in the composite end point for patients who received the higher doses of canakinumab (150mg or 300mg every 3 months) in the median follow-up time of 3.7 years.^15^ Whether the effects of canakinumab could modify the evolution of PAD is unknown. However, the common pathways of atherosclerosis and inflammation are reproduced in different sites of arterial disease, leading to the promise that PAD individuals could have benefits with such therapy.

Inclacumab directly links to P-selectin, a cell adhesion molecule, inhibiting its inflammatory and thrombotic effects.^16^^,^^17^ Inclacumab expressively reduced myocardial injury in certain types of acute myocardial infarction.^18^ Also, P-selectin was pointed as an independent risk factor for PAD progression and ankle-brachial index reduction.^19^ Although this rationale suggests that P-selectin antagonism by inclacumab could theoretically change the evolution of PAD, no clinical investigation was yet done for such purpose.

In conclusion, we can see evidences of an important change in the scenario of clinical therapy of peripheral artery disease in the present and in a near future. These new therapeutic possibilities will change the fate of PAD patients in all stages of their disease, with a very likely impact in the future of surgical/interventional therapy.
